# Risk factors and outcomes of emerging *Listeria monocytogenes* infection in Pakistan: Insights from a tertiary care hospital

**DOI:** 10.1371/journal.pone.0346612

**Published:** 2026-04-22

**Authors:** Zara Nadeem, Iffat Khanum, Imran Ahmed, Tahir Munir, Kiren Habib

**Affiliations:** 1 Department of Medicine, Akuh, Pakistan; 2 Department of Pathology and Laboratory Medicine, Akuh, Pakistan; Purdue University, UNITED STATES OF AMERICA

## Abstract

**Background:**

*Listeria monocytogenes* is a Gram-positive rod responsible for listeriosis. It has emerged as a significant foodborne pathogen and has been implicated in numerous outbreaks worldwide often present with bacteremia and may progress to severe manifestations such as meningoencephalitis, particularly in immunocompromised individuals and the elderly. Maternofetal infection is associated with worse pregnancy outcomes. The objective of this study is to assess the risk factors, clinical features, and outcomes of patients admitted with *Listeria monocytogenes* infection at a tertiary care Centre in Karachi, Pakistan.

**Methods:**

A retrospective study was conducted over 7 years and included all patients with culture-proven listeriosis. Comorbid conditions, clinical presentation, treatment, and outcomes were recorded and analyzed.

**Results:**

A total of 63 patients diagnosed with *Listeria monocytogenes* infection were included in the study. There was a female predominance (n = 44, 69.8%), with a median age of 53**(32.0, 67.0)** years. Diabetes mellitus was the most common comorbidity (n = 27, 42.9%). Among high-risk groups, 14 (22.6%) patients were pregnant, 10 (15.9%) were on immunosuppressive therapy, and another 10 (15.9%) were classified as elderly. The predominant presenting symptoms included fever (n = 47, 74.6%) and central nervous system involvement (n = 28, 44.4%), mainly meningoencephalitis. All patients received antibiotic therapy with either ampicillin or meropenem for a mean duration of 16.7 ± 8.4 days. The overall mortality rate was11.1%.(n = 7).

**Conclusion:**

Listeriosis was observed not only in the elderly but also in middle-aged individuals with underlying risk factors, as well as in pregnant women.Improved environmental hygeine, early diagnosis, and timely treatment are essential to improving outcomes, particularly in pregnancy-related cases. Public education, healthcare provider training, and community-level preventive strategies are critical for effective management and control of listeriosis.

## Introduction

*Listeria monocytogenes* is a Gram-positive rod, known to be an opportunistic food-borne pathogen worldwide.[1] Its most common route of transmission in the community is consumption of contaminated food.[2] *L. Monocytogenes* is capable of persisting under adverse conditions, including low moisture, refrigeration temperatures, and high-salt environments, which pose significant challenges for its control in food systems.[3] Due to its prolonged incubation period, prompt diagnosis is difficult. Numerous reports of listeriosis outbreaks have been linked to dairy & meat products, as well as fresh produce [4]It is now widely acknowledged to be a major human pathogen, capable of causing not only non-invasive infections but also severe and often fatal illnesses, notably meningoencephalitis and maternal–fetal infections [5].The annual incidence rate of listeriosis varies from 0.1 to 10 cases per million people per year, depending on the countries and regions of the world [6].In Southeast Asia, the prevalence is estimated at 22.2% [7]. Only a limited number of studies on *Listeria monocytogenes* in humans have been conducted in Pakistan, with most existing research focusing on the agricultural sector. In 2022, a case series reported an increasing number of cases, underscoring the need for strategic planning to address this growing concern.[8]

Ready-to-eat dairy, seafood, and immunocompromised individuals, including pregnant women and geriatric populations, seem to be most affected by this pathogen [9–11].This pathogen is frequently associated with ready-to-eat dairy and seafood products [9,11]. In humans, the highest burden of disease is observed among immunocompromised individuals, particularly pregnant women and older adults [10].Despite the low prevalence, it has a high mortality rate, particularly in high-risk populations such as neonates, pregnant females, age ≥ 60 years, primary bacteremia, CNS involvement and prior comorbidities such as malignancies, chronic kidney diseases [12]. Maternal infections with *Listeria monocytogenes* have been reported to be rare before 20 weeks of gestation. The incidence of listeriosis is higher among pregnant women than in the general population and is associated with adverse fetal outcomes, including miscarriage and stillbirth [11,13].

Treatment recommendations for listeriosis rely on knowledge gained from animal models, in vitro experiments, and clinical studies, as listeriosis is not a common disease and there aren’t many effective randomized controlled trials. Ampicillin is the recommended treatment. whereas trimethoprim/sulphamethoxazole and meropenem are alternative agents in case of penicillin allergy [14]. Although penicillin and ampicillin are the recommended treatments for listeriosis, most experts advise combining gentamicin with ampicillin due to its delayed bactericidal activity against *Listeria* [15]. While some retrospective studies have failed to show a survival benefit—and even suggested potential harm in neurolisteriosis—other data indicate a possible survival advantage when gentamicin is administered for more than three days [16].

In Pakistan, published data on listeriosis remains limited. Until recently, most available evidence consisted of isolated case reports. A recent retrospective study from Pakistan described the clinical features, risk factors, and outcomes of culture-confirmed listeriosis over a five-year period, reporting substantial morbidity and adverse fetal outcomes. However, data comparing the clinical spectrum of listeriosis with respect to patient subgroups, comorbidities and outcomes are still scarce.[17] In regions with a high burden of non-communicable diseases, especially diabetes, the risk factors profile and clinical spectrum of disease may differ from previous studies conducted in developed countries.

We aimed to identify the risk factors, clinical characteristics, and outcomes of listeriosis at a tertiary care centre in Pakistan, to understand the various clinical manifestations and predisposing conditions in our population to facilitate early recognition and management of this potentially treatable condition.

## Materials and methods

This is a retrospective observational study including patients from January 2017 to December 2023, with medical records reviewed between January and December 2024 at the Aga Khan University Hospital (AKUH), Karachi, Pakistan. AKUH is a Joint Commission International (JCI) accredited, state-of-the-art tertiary care center that provides treatment to patients from across the country. All patients admitted with confirmed *L. monocytogenes* were evaluated for possible inclusion in the study. *Listeria monocytogenes* was detected either by culture in various specimens or by a commercial multiplex PCR assay in cerebrospinal fluid. The susceptibility testing was performed and interpreted according to the European Committee on Antimicrobial Susceptibility testing (EUCAST). [18] Patients with unrelated infections were excluded from the study; however, individuals with documented bacterial co-infections were included in the analysis. Patients were identified through the microbiology laboratory database, and then their electronic and paper-based medical records, along with radiology and other laboratory records were reviewed. Relevant information—including demographics, comorbidities, predisposing factors, immune status, radiological findings, management strategies, complications, and clinical outcomes—was recorded on a structured proforma.

### Ethical consideration

The study was reviewed and approved by the Ethics Review Committee, Aga Khan University (ERC # 2023-9294-26813). Due to the retrospective chart reviews and lack of direct involvement of patients or other human participants, a waiver of informed consent was given by the Ethics Review Committee, Aga Khan University.

Data analysis was performed using RStudio (version 4.1.2; Boston, USA). The Shapiro-Wilk test was used to assess the normality of quantitative variables, including age, premature birth, and gestational age. As all variables were found to be non-normally distributed, they were summarized using median and interquartile range (IQR). Categorical variables, such as gender, comorbidities, clinical manifestations, and patient outcomes, were summarized as frequencies and percentages. Stratified analysis was conducted based on the diagnosis, i.e., Neurolisteriosis or Non-neurolisteriosis, to explore potential statistical associations. The Mann-Whitney U test was used for continuous variables, while the Chi-square or Fisher’s exact test was applied for categorical variables, as appropriate.

## Results

A total of 63 patients met the inclusion criteria and were included in the study. The mean age of patients was 53 [Q1, Q3] [32.0, 67.0] years, and the majority were female (n = 44, 69.8%). Diabetes Mellitus (n = 27, 43.5%) was the most frequent comorbidity, followed by hypertension (n = 25, 40.3%) and (n = 12, 19.0%) (Table 1). Among the high-risk group, 14 (22.6%) patients were pregnant, 10 (15.9%) were on immunosuppressive therapy, and a similar percentage was classified as elderly patients (Table 2).

**Table 1:** Demographic characteristics and comorbid conditions of patients with listeriosis (n = 63).

Variable	n (%) or Median (Q1, Q3)
**Age (years)**	**53.0 (32.0, 67.0)**
**Gender**	
**Male**	**19 (33.2)**
**Female**	**44 (69.8)**
**Comorbid conditions**	
**Hypertension**	**25 (39.7)**
**Diabetes mellitus**	**27 (42.9)**
**HIV positive**	**1 (1.6)**
**Autoimmune disease**	**7 (11.1)**
**Malignancy**	**6 (9.5)**
**Chronic kidney disease**	**5 (7.9)**
**Chronic liver disease**	**12 (19.0)**
**Other known diseases**	**30 (47.6)**
**Clinical risk factors**	
**Immunocompromised**	**17 (27.0)**
**On immunosuppressive therapy**	**10 (15.9)**
**Pregnancy**	**14 (22.2)**
**Extremes of age**	**10 (15.9)**
**Alcoholism**	**2 (3.2)**
**Hypothyroidism/ hemolytic anemia**	**1 (1.6)**
**Multiple myeloma**	**1 (1.6)**

Data are presented as n (%) unless otherwise specified. Age is presented as median (Q1, Q3).

**Table 2:** Clinical Manifestation of patients with Listeria Monocytogenes infection.

Clinical Manifestation	Number of Patients (n)	Percentage (%)
**Diagnosis**		
CNS Listeria	28	44.4
Non-CNS listeria	35	55.6
**Breakdown of CNS Listeria**		
Meningoencephalitis	27	96.4
Brain Abscess	1	3.6
**Breakdown of Non-CNS Listeria**		
Bacteremia	15	42.9
Colitis Abdominal infections (colitis/ peritonitis)	3	8.6
Placental Infection	3	8.6
Urinary tract infection	1	2.8
Non-specific diagnosis	−13	37.1
**Presenting Symptoms**		
Fever	47	74.60
Drowsiness	20	31.74
Headache	8	12.7
Seizure	7	11.11
Abdominal Pain	10	15.9
Vomiting	9	14.3
Diarrhea	4	6.34
Other nonspecific symptoms- flu, jaundice, Per rectal bleeding, dysphagia	12	19.047
**PATIENTS OUTCOME**		
Discharged	44	69.8
Lost to follow up	12	19.1
Expired	7	11.1

The most common clinical features were fever (n = 47, 74.6%), followed by drowsiness (n = 20, 31.7%), abdominal pain and vomiting (n = 10, 15.9%). CNS listeriosis was found in 28(44.4%) Patients presented with listeriosis, predominantly meningoencephalitis; one patient had a brain abscess, and 16 (25.39%) patients had concomitant bacteremia as well (Table 3).

**Table 3:** Straification analysis based on diagnosis.

Variable	CNS Listeria(N = 28)	Non-CNS Listeria (N = 35)	P-value
**Age (years)**			0.254
Median [Q1, Q3]	58.5 (36.5, 68.0)	50.0 (28.0, 65.0)	
**Gender**			0.179
Female	17 (60.7%)	27 (77.1%)	
Male	11 (39.3%)	8 (22.9%)	
**Hypertension**	13 (46.4%)	12 (34.3%)	0.438
**Diabetes**	14 (50.0%)	13 (37.1%)	0.321
**HIV**	1 (3.6%)	0 (0.0%)	0.444
**Autoimmune Disease**	3 (10.7%)	4 (11.4%)	1.000
**Malignancy**	4 (14.3%)	2 (5.7%)	0.393
**CKD**	3 (10.7%)	2 (5.7%)	0.648
**CLD**	6 (21.4%)	6 (17.1%)	0.752
**Other known diseases**	18 (64.3%)	12 (34.3%)	0.024
**Immunosuppression**	8 (28.6%)	9 (25.7%)	1.000
**On Immunosuppressive Agents**	6 (21.4%)	4 (11.4%)	0.318
**Pregnancy**	1 (3.6%)	13 (37.1%)	0.002
**Extreme of Age**	3 (10.7%)	7 (20.0%)	0.490
**Socioeconomic Status**			0.055
Low	1 (3.6%)	1 (2.9%)	
Middle	13 (46.4%)	26 (74.3%)	
High	14 (50.0%)	8 (22.9%)	
**Other Factors**			0.846
Alcohol use	1 (3.6%)	1 (2.9%)	
Multiple myeloma	1 (3.6%)	0 (0.0%)	
Hypothyroidism/ Hemolytic anemia	0 (0.0%)	1 (2.9%)	
None	26 (92.9%)	33 (94.3%)	
**Patient Outcome**			0.299
Discharged	19 (67.9%)	25 (71.4%)	
Expired	5 (17.9%)	2 (5.7%)	
Lost to follow-up	4 (14.3%)	8 (22.9%)	

Overall, antimicrobial susceptibility data were available for 59 cases. In three cases where *Listeria monocytogenes was* detected by a commercial multiplex PCR (Film array meningitis encephalitis panel, BioMérieux) the organism did not grow on culture. The susceptibility rates for ampicillin, meropenem and trimethoprim/sulphamethoxazole were 96.6%, 100% and 86.4% respectively.

All patients were treated with ampicillin or meropenem, depending upon the availability of drugs for 4–6 weeks (mean 16.7 ± 8.4) days. The overall mortality was 11.1% (n 5/7) and most of the patients who died were older than 60 years of age

In subgroup analysis between CNS and non-CNS listeriosis (Table 3), there was no major difference, i.e., age, comorbid illness and gender distribution and mortality. Pregnancy was significantly more common in the non-CNS group (37.1% vs. 3.6%, p = 0.002). Mortality was observed in 5 patients (15.2%) in CNS listeriosis and 2 patients (6.7%) in the non-CNS group with no statistically significant difference observed between two groups (p value 0.299). Pregnancy-related poor fetal outcome was significantly higher in non-CNS involvement, with 5 patients (16.7%) compared to 2 patients (6.1%) in CNS listeriosis (p = 0.024).

## Discussion

Our study found that listeriosis predominantly affected middle-aged adults, with a predominance of female gender and a high prevalence of diabetes mellitus among the study population. Central nervous system involvement was observed in a considerable proportion of cases. Overall mortality was low, largely confined to elderly patients. Comparative analysis showed no significant differences between CNS and non-CNS disease; however worst pregnancy outcomes were significantly higher in non-CNS listeriosis among pregnant patients.

Listeriosis is more common among patients with prior risk factors like pregnancy, immunosuppressive state or advanced age.A higher incidence of *L. monocytogenes* infection has been reported in literature among individuals over 65 years of age, likely attributed to a weakened immune response and reduced ability to combat infectious agents [19,20]. The median age of our study population was 53 years, with only 15.9% of patients being more than 65 years of age. Yan Liu et al. also found a median age of 56 years, but with 40.7% patients aged ≥60 years.[21] In developing countries like Pakistan, increased cases of listeriosis among patients under 65 years of age may be secondary to poor food safety, inadequate sanitation, limited public awareness, weak health infrastructure and challenges in disease surveillance, diagnosis and prevention. [22,23]

Pakistan faces a growing burden of diabetes mellitus (DM), increasingly affecting younger individuals [24,25].The most recent estimate showed that 33 million people are living with diabetes in Pakistan, making it 3rd largest diabetes population globally. The overall prevalence of Diabetes is 26.7% among adults aged 20–70 years [26]. Studies have reported the increasing prevalence of diabetes among middle age and younger patients among Pakistani population. [27]DM impairs immune responses, predisposing patients to infections. They have 1.5- to 4-fold increased risk of infections and are associated with poor outcome [27]. Hyperglycemia promotes bacterial growth, enhances bacterial virulence and impairs host immune dysfunction. In addition, diabetic neuropathy and vascular insufficiency further predispose individuals to the development of infections [28]The relatively lower median age group observed in our cohort may be attributable to the high prevalence of diabetes,which increases the susceptibility of infections irrespective of age, in contrast to usual predilection of listeria for older adults.

Pregnant women are at increased risk of developing listeriosis than the general population, with an increased risk of severe disease, complications and adverse obstetric outcomes. This is consistent with our study, in which 50% of pregnancies resulted in adverse outcomes, including intrauterine death, miscarriages or neonatal death. A recent review on listeriosis during pregnancy also highlighted the significant adverse impact of *Listeria* infection on pregnancy outcomes, including stillbirth, intrauterine fetal demise, spontaneous abortion, neonatal listeriosis, and preterm birth. *L monocytogenes* exhibits a marked affinity for the placental tissues, enabling transplacental transmission, subsequent fetal infection and adverse pregnancy outcomes despite prompt medical treatment. Maternal listeriosis has been associated with miscarriages stillbirth across all gestational ages [29]. Available evidence suggests that pregnancy does not independently increase the risk of neurolisteriosis and CNS involvement in pregnant women without underlying immunodeficiency is extremely rare [30–32]. None of the pregnant patients in our study had an underlying immunosuppressive condition. Another possible explanation of few pregnant patients with neurolisteriosis in our study could be early initiation of antibiotic treatment.CNS involvement is usually secondary to hematogenous spread. In pregnant women, listeriosis often presents with non-specific systemic symptoms, prompting earlier healthcare-seeking behavior and earlier initiation of antibiotic therapy. Timely treatment may limit hematogenous dissemination to the central nervous system, thereby reducing the likelihood of CNS involvement. This underscores the importance of educating pregnant women about preventive measures, raising awareness among healthcare professionals, including obstetricians, regarding early diagnosis and management of listeriosis during pregnancy to improve maternal and fetal outcomes.

Neurolisteriosis was also common in our study, with most patients experiencing meningitis or meningoencephalitis, and only one patient presenting with a brain abscess. The MONALISA group also reported that, among patients with neurolisteriosis, meningoencephalitis was the predominant presentation (84%), and brainstem involvement was observed in only 17%. In the subgroup analysis, no statistically significant difference was found between patients with and without CNS involvement with regards to demographic characteristics, comorbidities, clinical presentations, and outcomes. CNS involvement appears to be less common among pregnant females. The incidence of neurolisteriosis reported in the literature is 30% but the occurrence of cerebral abscess secondary to *L. monocytogenes* is only 3% [33].Our patient with L*isteria* brain abscess was an elderly female with a history of diabetes mellitus, hypertension, non-B non-C chronic liver disease and showed complete recovery after 6 weeks of antibiotics.There is limited evidence available, primarily derived from case reports, about the management of *Listeria* brain abscess. Prolonged antibiotic therapy (5–6 weeks) appears reasonable, with variable results of adjunctive surgical drainage [34–36]

A recent meta-analysis reported a listeriosis-related mortality rate of approximately 23%, with most deaths occurring in individuals over 60 years of age and found that gender has no impact on mortality risk [12]. The risk factors of mortality associated with listeriosis are neurolisteriosis, prior malignancies, pulmonary diseases, chronic kidney diseases, cardiovascular diseases, immunosuppression and multi-organ failure [5,12,37].The lower mortality observed in our study may be explained by differences in patient demographics and clinical characteristics compared with the referenced meta-analysis. While the meta-analysis predominantly included elderly patients (≥60 years) with multiple high-risk comorbidities known to increase listeriosis-related mortality, our cohort largely comprised middle-aged individuals with a lower burden of comorbid illness except diabetes. Given the well-established association between advanced age, multimorbidity, and poor outcomes in listeriosis, these differences likely contributed to the reduced mortality in our population. Additionally, management at a single tertiary-care center with early diagnosis, timely initiation of appropriate antimicrobial therapy, and consistent access to comprehensive supportive care may have further improved patient outcomes.

We also found high mortality among the elderly population, with no significant difference between males and females. All except one had existing comorbid illness or immunosuppressive state, but neurolisteriosis was not identified as a risk factor of mortality in the current study. This might be due to small sample size and timely management of patients presenting with neurological involvement, i.e., prompt diagnostic evaluation and early initiation of antibiotics in this high-risk group.

Our study has several limitations. It is a single-center, retrospective study with a small sample size, and large-scale multicenter studies are required to validate our findings. It was a hospital-based study; many patients with less severe infections may have been managed in primary health centers and might not have presented to tertiary care hospitals, potentially leading to under representation of milder cases. Additionally, retrospective design may have resulted in incomplete documentation with missing information on predisposing factors such as dietary history and environmental exposures.

## Conclusion

Listeriosis significantly affects not only the elderly but also middle-aged individuals, particularly those with underlying risk factors and during pregnancy. Environmental hygiene, early diagnosis, and prompt treatment are crucial to reducing adverse outcomes, especially in pregnancy-related cases. Raising public awareness, educating healthcare workers for early diagnosis and management, and implementing preventive measures at the community level can improve listeriosis outcomes.

## References

[1] Maertens De Noordhout C, Devleesschauwer B, Maertens De Noordhout A, Blocher J, Haagsma JA, Havelaar AH, et al. Comorbidities and factors associated with central nervous system infections and death in non-perinatal listeriosis: a clinical case series. BMC Infect Dis. 2016;16:256. doi: 10.1186/s12879-016-1602-3 27267465 PMC4897813

[2] Feng Y, Wu S, Varma JK, Klena JD, Angulo FJ, Ran L. Systematic review of human listeriosis in China, 1964-2010. Trop Med Int Health. 2013;18(10):1248–56. 24016031 10.1111/tmi.12173

[3] Capita R, Felices-Mercado A, García-Fernández C, Alonso-Calleja C. Characterization of Listeria Monocytogenes Originating from the Spanish Meat-Processing Chain. Foods. 2019;8(11):542. doi: 10.3390/foods8110542 31684121 PMC6915328

[4] Ravindhiran R, Sivarajan K, Sekar JN, Murugesan R, Dhandapani K. Listeria monocytogenes an emerging pathogen: a comprehensive overview on listeriosis, virulence determinants, detection, and anti-listerial interventions. Microb Ecol. 2023;86(4):2231–51. 37479828 10.1007/s00248-023-02269-9

[5] Charlier C, Perrodeau É, Leclercq A, Cazenave B, Pilmis B, Henry B, et al. Clinical features and prognostic factors of listeriosis: the MONALISA national prospective cohort study. Lancet Infect Dis. 2017;17(5):510–9. doi: 10.1016/S1473-3099(16)30521-7 28139432

[6] Zahid R, Arbab Z, Tahir Z, Tehseen U, Ali S, Bukhsh SK, et al. Global Prevalence of Listeriosis. Faisalabad. 2023.

[7] Chaudhry S. Higher prevalence of Listeriosis in Indian subcontinent, a food related menace. Int J Pharm Chem Anal. 2023;10:1–2.

[8] Irshad M, Jamil B. Listeria monocytogenes outbreak – serendipity or emerging threat?. Infect Dis J Pakistan. 2022;31(2 SE-Original Article):49–52.

[9] Preußel K, Milde-Busch A, Schmich P, Wetzstein M, Stark K, Werber D. Risk factors for sporadic non-pregnancy associated listeriosis in germany-immunocompromised patients and frequently consumed ready-to-eat products. PLoS One. 2015;10(11):e0142986. doi: 10.1371/journal.pone.0142986 26599484 PMC4658106

[10] Leclercq A, Kooh P, Augustin J-C, Guillier L, Thébault A, Cadavez V, et al. Risk factors for sporadic listeriosis: A systematic review and meta-analysis. Microbial Risk Analysis. 2021;17:100128. doi: 10.1016/j.mran.2020.100128

[11] Ke Y, Ye L, Zhu P, Sun Y, Zhu Z. Listeriosis during pregnancy: a retrospective cohort study. BMC Pregnancy Childbirth. 2022;22(1):261. doi: 10.1186/s12884-022-04613-2 35346105 PMC8962181

[12] Huang C, Lu T-L, Yang Y. Mortality risk factors related to listeriosis - A meta-analysis. J Infect Public Health. 2023;16(5):771–83. doi: 10.1016/j.jiph.2023.03.013 36958172

[13] Kuang L, Lai Y, Gong Y. Analysis of listeriosis infection cases during pregnancy among 70 131 deliveries. J Obstet Gynaecol Res. 2022;48(1):66–72. doi: 10.1111/jog.15063 34657360

[14] Hof H. Therapeutic options. FEMS Immunol Med Microbiol. 2003;35(3):203–5. doi: 10.1016/s0928-8244(02)00466-2 12648838

[15] Temple ME, Nahata MC. Treatment of Listeriosis. Ann Pharmacother. 2000;34(5):656–61. doi: 10.1345/aph.19315 10852095

[16] Sutter JP, Kocheise L, Kempski J, Christner M, Wichmann D, Pinnschmidt H, et al. Gentamicin combination treatment is associated with lower mortality in patients with invasive listeriosis: a retrospective analysis. Infection. 2024;52(4):1601–6. doi: 10.1007/s15010-024-02330-w 38963609 PMC11289180

[17] Zaheer F, Usman M, Raza S, Chaudhary NN, Babar W, Rahat A, et al. Listeriosis - a retrospective study of 5 years on risk factors and clinical outcomes at a tertiary care hospital in Islamabad, Pakistan. Access Microbiol. 2026;8(2):001069.v3. doi: 10.1099/acmi.0.001069.v3 41647795 PMC12872326

[18] European Committee on Antimicrobial Susceptiblity Testing. Breakpoint tables for interpretation of MICs and zone diameters. EUCAST [Internet]. Available from: https://www.eucast.org/fileadmin/eucast/pdf/breakpoints/v_16.0_Breakpoint_Tables.pdf

[19] Pohl AM, Pouillot R, Bazaco MC, Wolpert BJ, Healy JM, Bruce BB, et al. Differences Among Incidence Rates of Invasive Listeriosis in the U.S. FoodNet Population by Age, Sex, Race/Ethnicity, and Pregnancy Status, 2008-2016. Foodborne Pathog Dis. 2019 Apr;16(4):290–7. 30735066 10.1089/fpd.2018.2548 PMC6482898

[20] Vital signs: Listeria illnesses, deaths, and outbreaks--United States, 2009-2011. MMWR Morb Mortal Wkly Rep. 2013;62(22):448–52. 23739339 PMC4604984

[21] Liu Y, Xie Y, Liu L, Wang J, Li W, Yang C, et al. Clinical, molecular, and resistance features of Listeria monocytogenes in non-perinatal patients with listeriosis: 8-year retrospective data from four tertiary hospitals in Shandong, China. PeerJ. 2025;13:e19126. doi: 10.7717/peerj.19126 40093420 PMC11908440

[22] Munir MZ, Khan JA, Ijaz M, Akhtar F. Molecular characterization and hematological analysis of Listeria monocytogenes infection in dairy cows in Punjab (Pakistan). Arch Microbiol. 2022;204(4):201. doi: 10.1007/s00203-022-02804-1 35239048

[23] Samad D. Isolation and identification of Listeria monocytogenes from raw vegetables and meat sold in Quetta Pakistan. Pak J Zool. 2019;52.

[24] Azeem S, Khan U, Liaquat A. The increasing rate of diabetes in Pakistan: A silent killer. Ann Med Surg (Lond). 2022;79:103901. doi: 10.1016/j.amsu.2022.103901 35860160 PMC9289249

[25] Amin F, Imran M, Hafeez SA, Zehra B. Diabetes and its associated factors: A Retrospective cohort analysis of a large database at Indus Hospital Health Network. Pak J Med Sci. 2024;40(2ICON Suppl):S10–4. doi: 10.12669/pjms.40.2(ICON).8948 38328649 PMC10844897

[26] Sun H, Saeedi P, Karuranga S, Pinkepank M, Ogurtsova K, Duncan BB, et al. IDF Diabetes Atlas: Global, regional and country-level diabetes prevalence estimates for 2021 and projections for 2045. Diabetes Res Clin Pract. 2022;183:109119. doi: 10.1016/j.diabres.2021.109119 34879977 PMC11057359

[27] Holt RIG, Cockram CS, Ma RCW, Luk AOY. Diabetes and infection: review of the epidemiology, mechanisms and principles of treatment. Diabetologia. 2024;67(7):1168–80. doi: 10.1007/s00125-024-06102-x 38374451 PMC11153295

[28] Darwitz BP, Genito CJ, Thurlow LR. Triple threat: how diabetes results in worsened bacterial infections. Infect Immun. 2024;92(9):e0050923. doi: 10.1128/iai.00509-23 38526063 PMC11385445

[29] Kraus V Jr, Čižmárová B, Birková A. Listeria in Pregnancy-The Forgotten Culprit. Microorganisms. 2024;12(10):2102. doi: 10.3390/microorganisms12102102 39458411 PMC11510352

[30] Wang Z, Tao X, Liu S, Zhao Y, Yang X. An update review on listeria infection in pregnancy. Infect Drug Resist. 2021;14:1967–78. 34079306 10.2147/IDR.S313675 PMC8165209

[31] Disson O, Lecuit M. Targeting of the central nervous system by Listeria monocytogenes. Virulence. 2012;3(2):213–21. doi: 10.4161/viru.19586 22460636 PMC3396700

[32] Lu B, Yang J, Gao C, Li D, Cui Y, Huang L. Listeriosis cases and genetic diversity of their L. monocytogenes isolates in China, 2008-2019. Front Cell Infect Microbiol. 2021;11:608352. 33680989 10.3389/fcimb.2021.608352 PMC7933659

[33] Boully A, Casenaz A, Blot M, Piroth L, Thai M, Zanetta G, et al. A brain problem with Listeria monocytogenes. Lancet Infect Dis. 2022;22(2):296. doi: 10.1016/S1473-3099(21)00683-6 35092802

[34] Carneiro BH, de Melo TG, da Silva AFF, Lopes JT, Ducci RD-P, Carraro Junior H, et al. Brain abscesses caused by Listeria monocytogenes in a patient with myasthenia gravis - Case report and systematic review. Infez Med. 2023;31(4):570–4. doi: 10.53854/liim-3104-16 38075415 PMC10705864

[35] Zhang C, Yi Z. Brain abscess caused by Listeria monocytogenes: a case report and literature review. Ann Palliat Med. 2022;11(10):3356–60. doi: 10.21037/apm-22-383 35695050

[36] Tiri B, Priante G, Saraca LM, Martella LA, Cappanera S, Francisci D. Listeria monocytogenes brain abscess: controversial issues for the treatment - two cases and literature review. Case Reports Infectious Diseases. 2018;2018:6549496. doi: 10.1155/2018/6549496 PMC6081550 30140475

[37] Xu W, Peng M-J, Lu L-S, Guo Z-J, Li A-M, Li J, et al. Clinical characteristics and fatality risk factors for patients with listeria monocytogenes infection: a 12-year hospital-based study in Xi’an, China. Infect Dis Ther. 2024;13(6):1359–78. doi: 10.1007/s40121-024-00986-3 38733495 PMC11128421

